# Insecticide resistance profiles for malaria vectors in the Kassena-Nankana district of Ghana

**DOI:** 10.1186/1475-2875-8-81

**Published:** 2009-04-23

**Authors:** Francis Anto, Victor Asoala, Thomas Anyorigiya, Abraham Oduro, Martin Adjuik, Seth Owusu-Agyei, Dominic Dery, Langbong Bimi, Abraham Hodgson

**Affiliations:** 1 Navrongo Health Research Centre, Navrongo Health Research Centre, Ghana Health Service, P. O. Box 114, Navrongo, Upper East Region, Ghana; 2 Kintampo Health Research Centre, P. O Box 200, Kintampo, Brong Ahafo Region, Ghana; 3 Department of Zoology, University of Ghana, Legon, Ghana

## Abstract

**Background:**

Malaria is a major public health problem in Ghana. The current strategy of the National Malaria Control Programme is based on effective case management and the use of insecticide treated bed nets among vulnerable groups such as children under-five years of age and pregnant women. Resistance to pyrethroids by *Anopheles gambiae s.l*. and *Anopheles funestus *has been reported in several African countries including neighbouring Burkina Faso.

**Methods:**

Indoor resting *Anopheles *mosquitoes were collected. Blood-fed and gravid females were allowed to oviposit, eggs hatched and larvae reared to 1–3 days old adults and tested against permethrin 0.75%, deltamethrin 0.05%, cyfluthrin 0.15%, lambdacyhalothrin 0.1% and DDT 4%, based on WHO methodology. PCR analyses were carried out on a sub-sample of 192 of the *An. gambiae *for sibling species complex determination. Resistance to pyrethroids and DDT was determined by genotyping the knock-down resistance kdr gene mutations in the study area.

**Results:**

A total of 9,749 1–3 days-old F1 female *Anopheles *mosquitoes were exposed to the insecticides. Among the pyrethroids, permethrin, 0.75% had the least knockdown effect, whilst cyfluthrin 0.15%, had the highest knock-down effect. Overall, no difference in susceptibility between *An. gambiae *93.3% (95% CI: 92.5–94.1) and *An. funestus *94.5% (95% CI: 93.7–95.3) was observed when exposed to the pyrethroids. Similarly, there was no difference in susceptibility between the two vector species (*An. gambiae *= 79.1% (95% CI: 76.6–81.8) and *An. funestus *= 83.5% (95% CI: 80.2–86.4) when exposed to DDT. Overall susceptibility to the insecticides was between 80% and 98%, suggesting that there is some level of resistance, except for cyfluthrin 0.15%. The kdr PCR assay however, did not reveal any kdr mutations. The analysis also revealed only the molecular M (Mopti) form.

**Conclusion:**

The findings in this study show that *An. gambiae *and *An. funestus*, the main malaria vector mosquitoes in the Kassena-Nankana district are susceptible to the insecticides being used in the treatment of bed nets in the malaria control programme. There is however, the need for continuous monitoring of the pyrethroids as the efficacy is not very high.

## Background

Malaria is a major public health problem in Ghana. The strategy of the National Malaria Control Programme is based on effective case management and the use of insecticide treated bed nets among vulnerable groups, such as children under five years of age and pregnant women.

There is renewed interest in the use of insecticides for malaria control because of the effectiveness of insecticide-treated materials that show promise in reducing malaria transmission and morbidity [1-3]. Insecticide-treated bed nets (ITN) have been used successfully in the Kassena-Nankana district of Ghana for over a decade now; first as the earliest experimental intervention trial followed by routine use among most community members. This led to the adoption of ITNs as national malaria control policy in support of the Roll Back Malaria (RBM) control programme.

The recent appearance of resistance in the malaria vector mosquitoes to the insecticides used for the treatment of bed nets in nearby countries is however a cause of concern in Ghana as this could impact negatively on the success of the current ITN programme. This is because vector susceptibility is a basic requirement for the efficacy of insecticides and for that matter the success of the RBM programme. Due to all year round irrigated agricultural activity and ITN use in the Kassena-Nankana district, it is logical that insect/vector resistance to the commonly used insecticides will develop with time. At present the insecticides for net-treatment are limited to the pyrethroids, as these are the only ones available and affordable. Any *Anopheles *strains that become resistant to any products of the compound often become cross-resistant to the others. Despite limited monitoring activities, resistances are already reported in a number of malaria vectors including some populations of *Anopheles gambiae *in Africa. The levels of resistance and impact on malaria control are not yet known.

The use of the pyrethroid insecticides is also spreading fast in public health due to its success stories in house-spraying, ITNs, insecticide-treated curtains, space spraying among others. Part of the debilitating effects of the wide use of pyrethroids is cross-resistance resulting from pyrethroids formulated for use in agriculture. This underscores the need to generate insecticide resistance related data that will be necessary to guide planning, implementation and evaluation of insecticides and their role in the control of malaria in Ghana.

The current study was to determine the resistance status of *An. gambiae *and *Anopheles funestus*, the main malaria vectors in the Kassena-Nankana district of northern Ghana. The assessment was carried out through the application of WHO Bioassay tests on the vectors and determination of any knock down resistance *(kdr*) genes that the vectors would have developed over the years. Investigations concentrated on four pyrethroids (permethrin, deltamethrin, lambdacyhalothrin and cyfluthrin) and DDT.

## Methods

### Study sites

The study was carried out in the Kassena Nankana District (KND) in northern Ghana. The district lies between 10°30' and 11°00'N, 1° 00' and 1° 30'W and borders Burkina Faso to the north. The size of the district is about 1,674 sq km of Sahelian savannah with a population of 143,000 (NDSS, 2006). This is the area, where a large-scale insecticide treated bed net trial was undertaken about a decade ago, and has high malaria attributable infant and child mortality rate [3].

Most of the people live in multi-family compounds, which form the basis of the address system used in the Navrongo Demographic Surveillance System (NDSS, 2006), and are separated from one another by agricultural land. Virtually all the inhabitants engage in subsistence farming of millet, groundnut, and livestock. The average annual rainfall is 850 mm, almost all of which occurs in the months of May to September, with the rest of the year being relatively dry. A large reservoir (Tono dam) in the middle of the district and about 90 dug-out dams provide water throughout the year for agricultural irrigation purposes.

### Field procedures

Mosquitoes were collected from all the ecological areas (irrigated, low land and rocky highland) in the district. Indoor resting mosquitoes were collected using hand held aspirators and kept in paper cups covered with mesh. Moist cotton balls were added to the collection cups to increase survival of the samples. The blood fed and gravid females *An. gambiae *and *An. funestus *were allowed to oviposit and the larvae bred into adults in Navrongo Health Research Centre insectary.

### Mosquito identification

Female *Anopheles *mosquitoes obtained in the field or reared in the laboratory were identified by morphological characteristics using the criteria of Gilles and de Meillon [4]. Mosquito species collected from the field were maintained in the insectary at 26°C and 80% Relative Humidity (RH). The 80% RH was maintained using a humidifier. Insecticide assays were conducted in a separate laboratory isolated from insect rearing areas.

### Laboratory procedures

#### WHO susceptibility tests

Laboratory-bred live mosquitoes were held for up to four hours before testing and supplied with cotton balls soaked with 10% sucrose solution. Susceptibility tests were performed using WHO test kits for measuring insecticide resistance. The insecticides tested were: permethrin 0.75%, deltamethrin 0.05%, cyfluthrin 0.15%, lambdacyhalothrin 0.1% and DDT 4%. Bioassays were done according to standard WHO methodology [5]. Female *An. gambiae *and *An. funestus *mosquitoes were exposed to insecticide-impregnated filter papers. Susceptibility tests were conducted using 1–3 days-old unfed female mosquitoes by exposing groups of 20–25 to established discriminating concentrations of each insecticide. Each experiment consisted of three replicates of the test and one control (filter paper without insecticide). The mosquitoes were exposed for a period of one hour with the assay cylinders in a vertical position. The number of mosquitoes knocked down after 10, 15, 20, 30 and 60 minutes were recorded. After one hour, the mosquitoes were transferred into holding containers and provided with cotton pads with 10% sucrose solution. Mortality was recorded after a 24-hour recovery period.

#### Molecular forms and *kdr *analysis

PCR analysis was carried out on a sub-sample each of the *An. gambiae *and *An. funestus *mosquitoes for sibling species complex determination. Resistance to pyrethroids was determined by genotyping the knock down resistance *kdr *gene mutations [6] in the study area. One leg of a single mosquito was placed in a sterile 1.5 ml micro-centrifuge tube and crushed in 15 μl of double distilled water. The homogenized leg was then centrifuged at 14,000 RPM for 1 minute. DNA extract (supernatant) was then used immediately in the PCR mixture for the kdr gene identification. The PCR mixture consisted of an initial volume of 15.475 μl of double distilled water. To provide nucleotides to the DNA strand during extension, 0.1 μl of each of four 10 mM dNTPs (deoxyribonucleoside triphosphate) was added to the double distilled water in the tube. These include dTTP, dCTP, dGTP, dATP. Primers Agd1, Agd2, Agd3 and Agd4 were then added followed by a PCR buffer containing MgCl_2_. The final reagent added was 0.125 μl of Taq polymerase. 0.5 μl of the DNA extract was then pipetted into a 0.2 ml microtube followed by 19.6 μl of the PCR mixture and then placed in a Thermal Cycler, PTC-0100 to run for about three and a half hours for amplification. The amplified products were then analysed by electrophoresis on 1.5% agarose gel and visualized by ethidium bromide staining under UV light.

### Analysis of data

All data were entered into a computer database at the Navrongo Health Research Centre. Results of all resistance tests were analysed for knock-down/time relationships. Analyses produced time-mortality data, which is a highly effective measure of insecticide resistance. Comparisons in terms of efficacy of the insecticides as well as susceptibility of the two vector species were made using 95% confidence intervals. The genotyped results were used to interpret the pyrethroid resistance levels. Data used for the analysis are for bioassays that all the control mosquitoes exposed to untreated papers survived and so no corrections were made using Abbott's formula.

### Ethical issues

The protocol was submitted to both the Navrongo Health Research Centre and Ghana Health Service ethical review committees for ethical consideration and a waiver was given. Verbal consent was sought from compound/household heads before in-door resting mosquitoes were collected from peoples' homes.

## Results

### WHO susceptibility tests

A total of about 9,749, 1–3 day-old F1 female *Anopheles *mosquitoes (*Anopheles gambiae*, 5,676 and *An. funestus *4,073) were exposed to established discriminating concentrations of pyrethroid insecticides (permethrin 0.75%, deltamethrin 0.05%, cyfluthrin 0.15%, and lambdacyhalothrin 0.1%) and DDT (4%) under laboratory conditions to determine their level of susceptibility. Over 60% of the malaria vector mosquitoes exposed was knocked down within an hour of exposure to the chemicals.

**Figure 1:**
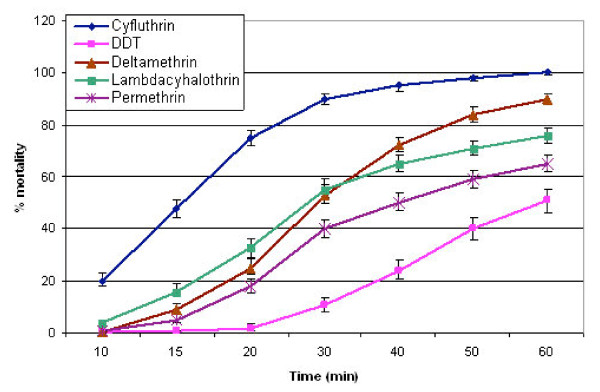
**Susceptibility of *Anopheles funestus *from the Kassena-Nankana district of northern Ghana to some pyrethroids and DDT**.

**Figure 2:**
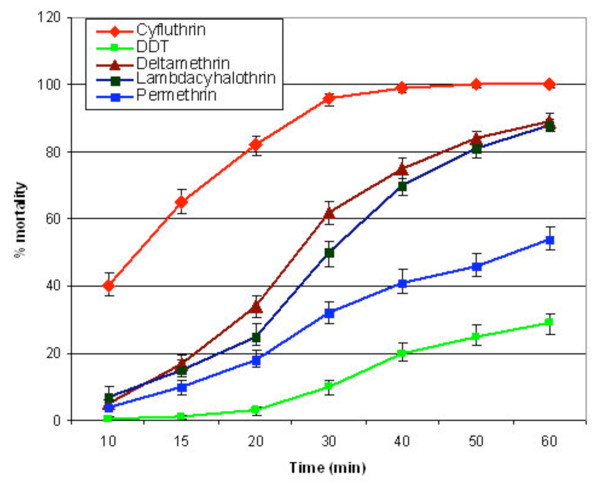
**The knock-down effect of some pyrethroids and DDT on *Anopheles gambiae *from the Kassena-Nankana district of Northern Ghana**.

Among the pyrethroids, permethrin, 0.75% had the least knock-down effect, 63.2% and 65.0% respectively for *An. gambiae *and *An. funestus*, whilst cyfluthrin 0.15%, had the highest knock-down effect (Table 1). The knock-down effect of cyfluthrin was significantly higher than the other three pyrethroids, whilst deltamethrin 0.05%, and lambdacyhalothrin 0.1%) were more effective than permethrin (Figures 1 and 2). Deltamethrin 0.05%, appeared to be slightly more potent (95.8%; CI: *94.9–96.6*) than lambdacyhalothrin 0.1% (93.6%; CI: 92.2–94.8) leading to slightly more mortalities in exposed *An. gambiae *and *An. funestus*. Overall, no difference in susceptibility between *An. gambiae *(93.3%; *CI: 92.5–94.1*) and *An. funestus *(94.5%; *CI: 93.7–95.3*) was observed when exposed to the pyrethroids. Similarly, the difference in susceptibility between the two vector species (*An. gambiae *= 79.1%; *CI: 76.6–81.8 *and *An. funestus *= 83.5%; *CI: 80.2–86.4*) was not statistically significant when exposed to DDT. The pyrethroids had a higher knocked-down effect and caused a higher level of mortality in the mosquitoes than DDT 4% which started knocking down the mosquitoes only after 20 minutes of exposure (Figures 1 and 2).

**Table 1:** Susceptibility of adult female *Anopheles gambiae *and *An. funestus *mosquitoes to selected pyrethroid insecticides and DDT

	*Number of mosquitoes tested*		**% knock-down after 1 hour exposure**	
	
Insecticides	*An. gambiae*	*An. funestus*	*An. gambiae*	*An. funestus*
Permethrin 0.75%	1372	881	63.2	65.0
Deltamethrin 0.05%	1451	1011	89.5	92.1
Cyfluthrin 0.15%	897	854	99.6	99.8
Lambdacyhalothrin 0.1%	888	758	88.2	94.4
DDT 4%	1068	569	33.8	50.6

### Susceptibility of mosquitoes collected from different ecological zones

The overall susceptibility of the malaria mosquitoes vectors collected from the different ecological areas to the insecticides Permethrin 0.75%, Deltamethrin 0.05%, Lambdacyhalothrin 0.1% and DDT 4.0% was between 80 and 98%. Susceptibility to Cyfluthrin however was much higher (Table 2).

**Table 2:** Mortality of adult female malaria mosquitoes vectors collected from different ecological areas of the Kassena-Nankana district when exposed to different insecticides

	Number of mosquitoes tested			% mortality		
	
Insecticide	Irrigated	Lowland	Rocky highland	Irrigated	Lowland	Rocky highland
Permethrin 0.75%	887	534	832	94.6	85.3	82.4
Deltamethrin 0.05%	1125	510	818	96.3	97.6	94.0
Cyfluthrin 0.15%	897	506	348	100	100	99.8
Lambda-cyhalothrin 0.1%	886	422	338	95.1	98.5	89.4
DDT 4%	765	512	360	82.7	77.5	85.9

### Seasonal variation in susceptibility of *An. gambiae *and *An. funestus *mosquitoes to selected pyrethroid insecticides and DDT

During the dry season, a total of 4,710 mosquitoes vectors (*An. funestus *and *An. gambiae *were exposed to the insecticides. Out this number, 4,432 (94.1%; CI: 93.4–94.8) were susceptible. A total of 5,039 were exposed during wet season with 4,527 (89.8%; CI: 89.0–90.7) being susceptible. Thus, overall, there was a significant seasonal variation in susceptibility. Analysis at the species level also revealed significant seasonal variation in susceptibility. For *An. funestus*, dry season susceptibility was 90.5% (*CI: 90.5–92.9*) and that for the wet season was 87.3% (*CI: 85.7–88.7*). In the case of *An. gambiae*, susceptibility during the dry season was 89.8% (*CI: 88.5–91.0*) and the wet season was 91.4% (*CI: 90.4–92.4*).

### Molecular forms and *kdr*

**Figure 3:**
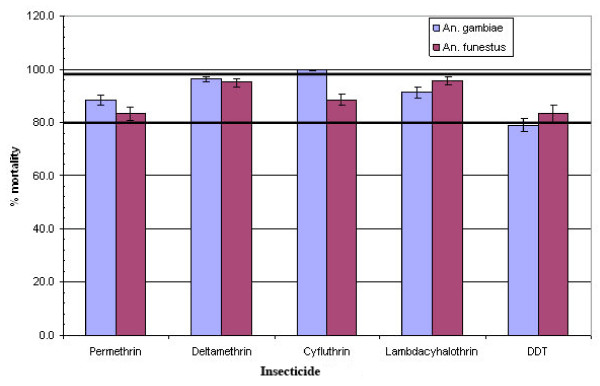
**Susceptibility of *Anopheles gambiae *and *Anopheles funestus *to pyrethroids and DDT**.

After allowing for a 24-hour recovery period, over 80% of the mosquitoes exposed died (Figure 3); mortality was however less than 98% (a level of mortality suggesting the possibility of resistance) except for cyfluthrin 0.15%. The *kdr *PCR assay of 192 of the samples (148 survivors (DDT: 67; deltamethrin: 17; permethrin: 64: and 44 susceptible ones) however, did not reveal any *kdr *mutations in the mosquitoes. The analysis also revealed only the molecular M (Mopti) form.

## Discussion

Female *An. gambiae *and *An. funestus *mosquitoes were collected from three micro-ecological areas (irrigated, low land and rocky highland) during the months of November 2006 to April 2007 (dry season) and July to October 2007 (wet season) in the Kassena-Nankana district of northern Ghana. These were bred in the laboratory and the F1 progeny exposed to deltamethrin 0.05%, lambdacyhalothrin 0.1%, permethrin 0.75%, cyflutrin 0.15%, and DDT 4%. Analysis of the results revealed that *An. gambiae *and *An. funestus *in the district are susceptible to the tested insecticides despite the over a decade of use of permethrin and deltamethrin in the treatment of bed nets in the district. Overall, between 80% and 98% of the exposed mosquitoes died during the 24-hour recovery period.

Susceptibility of *An. gambiae *collected from the low land and rocky highland areas was however less than 80%, suggesting resistance in this malaria vector species to DDT, an insecticide that was in common use in the 1950s and 60s. Resistance in *Anopheles gambiae *to pyrethroid insecticides has mainly been associated with reduced target site sensitivity arising from a single point mutation in the sodium channel gene, often referred to as knockdown resistance (*kdr*). PCR analysis of both samples that survived the sensitivity test and those that died however did not reveal the presence of the kdr gene [7]. Possibly, other mechanisms of resistance e.g. metabolic detoxification might be playing a role in the apparent resistance of *An. gambiae *collected from these micro-ecological areas. The knock down rate of DDT was also found to be slow as mosquitoes started getting knocked-down only after 20 minutes of exposure [8,9].

The low efficacy of permethrin and lambdacyhalothrin can be explained in terms of the extensive domestic and agricultural use of these two insecticides in the district [10,11]. There is also evidence of cross-resistance between DDT and the pyrethroids in *An. gambiae *[11], which can explain the low level of efficacy of both DDT and permethrin in our study area. Though DDT is no more in common use in the area, permethrin and other pyrethroids have been in use for a long time in agriculture for the control of pests and in the health sector for malaria control [3].

*Anopheles funestus *in the current study was found to be more susceptible to the insecticides during the dry season than during the wet season. Such seasonal variations in susceptibility of *Anopheles *mosquitoes to pyrethroids and DDT have been reported in neighbouring Burkina Faso. The increase in resistance of *An. gambiae *complex to permethrin 1%, DDT 4% and deltamthrin 0.05% in the wet season was explained in terms of increased use of insecticides on cotton farms resulting in selection pressure on the mosquito population [10]. In addition to selection pressure due to increased use of insecticides, the harsh dry season in the Kassena-Nankana district and neighbouring Burkina Faso may impact on the physiology of the insects and increase mortality when exposed to given concentrations of insecticides during that period of the year.

The M and S forms of the *An. gambiae *complex have been reported to live sympatrically in the Kassena-Nankana district [12]. This study however, found only the molecular M form of *An. gambiae *s.s. and without the *kdr *mutation. A study by Diabate and colleagues in neighbouring Burkina Faso also did not reveal any kdr mutation in this form of *An. gambiae *[10]. The dominance of the molecular M form however seems to correlate with ecological or climatic factors in the study area as the M form is more adapted to dryer environment and breeds along irrigated fields while the S form is normally found in humid forested areas and temporary pools [13]. The presence of the S and M forms is also known to change with the seasons [10].

Resistance of *An. gambiae *in West Africa to DDT has been known for a long time now [14,15]. It is expected, however, that after a long period of discontinuation of use, the efficacy will improve and could be used for indoor residual spraying since in costs less and has longer residual effect than the pyrethroids. The efficacy at the moment however, may not be good enough for use in a malaria control programme. The resistance of *Anopheles *species to the pyrethroids, the main insecticide being used by malaria control programmes for the treatment of bed nets has been reported in a number of African countries including Benin, Cote d'Ivoire, Burkina Faso, Kenya, Nigeria, Cameroun and Equatorial Guinea [9,10,16-18] 22 [19,20].

The findings in this study show that *An. gambiae *and *An. funestus*, the main malaria mosquitoes vectors in the Kassena-Nankana district [12] are susceptible to the insecticides being used in the treatment of bed nets in the malaria control programme. There is however, the need for continuous monitoring of the pyrethroids as the efficacy is not very high. This also holds for DDT.

## Competing interests

The authors declare that they have no competing interests.

## Authors' contributions

FA, VA, TA, AO and SOA designed the study, developed the instruments, took part in data analysis and manuscript production. AH contributed to the development of the protocol. DD contributed to the laboratory analysis of samples. MA took part in the data analysis. LB contributed to producing the Manuscript.
